# Supplementary material for: Are carbon emissions trading and green financial instruments synergistic? -Comprehensive quantitative research based on content analysis
Source: PLoS One. 2024 Mar 7;19(3):e0298601. doi: 10.1371/journal.pone.0298601 (PMC10919601; doi:10.1371/journal.pone.0298601)
Supplement: S1 Data — (ZIP) [file pone.0298601.s001.zip › Data/地级市Z_F工作报告.zip!/╕╩╦α/╛╞╚¬╩╨/└┤╫╘╣1⁄2╓┌║┼í╛┬φ┐╦╩2╛▌═°í┐_2014.docx]

各位代表：

　　现在，我代表酒泉市人民政府向大会作工作报告，请予审议，并请各位政协委员和其他列席人员提出意见。

　　2013年政府工作回顾

　　2013年，面对复杂多变的经济形势，全市各族人民深入贯彻党的十八大和十八届二中、三中全会、省第十二次党代会，以及市委三届三次、四次全委会议精神，认真落实省委、省政府和市委各项决策部署，按照“五做五加强”总体工作思路，强力推进“3341”项目工程，着力打造发展战略平台，努力营造良好发展环境，圆满完成了年初确定的各项目标任务。

　　——经济平稳较快增长。预计，全市完成生产总值659亿元，增长16%；财政总收入85.4亿元，财政总支出111.6亿元，分别增长18.3%和11.7%；公共财政预算收入26.7亿元，增长26.1%。

　　——结构调整步伐加快。设施农业快速发展，特色产业占农民人均纯收入的64%；新能源及装备制造首位产业规模扩大，对规模以上工业增长的贡献达到42.1%；文化、旅游产业蓬勃发展，现代服务业步伐加快，第三产业比重提高0.4个百分点。

　　——投资消费高位突破。“3341”项目工程深入推进，开工建设重大项目2264个，其中亿元以上项目332个；完成固定资产投资1058亿元，增长35%；社会消费品零售总额144亿元，增长18%。

　　——招商外贸成效显著。开工建设各类招商引资项目858个，引进到位资金886.3亿元，增长35%；实现外贸进出口总额8800万美元，增长20.5%。对外交流进一步扩大，与西安市签署建设丝绸之路经济带战略合作框架协议。

　　——人民生活持续改善。城镇登记失业率控制在3%，居民消费价格指数控制在103.5。实现城镇居民人均可支配收入23270元，农民人均纯收入11190元，分别增长16%；移民人均纯收入3426元，增长29.3%。政府承诺的校安工程、保障房建设、集中供热等惠民实事全面完成，各项约束性指标控制在省定目标以内。

　　1、发展壮大现代农业。制定实施“365”现代农业发展意见和农民收入倍增计划，整合使用财政强农支农专项资金，力促农业转型和农民增收，完成第一产业增加值75.2亿元，增长6.2%。打造农业发展战略平台，大力推进肃州国家级现代农业示范区和敦煌省级现代农业示范区建设，新建百亩连片日光温室示范区143个，新增设施高效农业12.5万亩，全市3000元以上高效田达到137万亩。深入推进牛羊大县建设，新增肉牛1万头、肉羊30万只。积极推进农业产业化经营，新增市级以上农民专业合作示范社40家，建成一批农业产业化项目，农产品加工增值率达到53.4%。创新农业发展方式，新增土地流转面积6万亩，培育500亩以上规模经营主体25个,实施标准化生产技术规程59项，建成农业科技示范园区66个。动物疫病防控措施全面落实，农产品质量安全抽检合格率达到99.5%。开展城乡劳动力职业技能培训3.56万人，输转劳动力18.6万人，实现劳务收入22.3亿元，增长15%。深入推进水权水价、集体林权、土地确权登记等农村改革，加强水利基础设施建设和生态治理，党河灌区生态治理和节水改造工程完成年度任务，发展高效节水11.4万亩，新增高标准基本农田6万亩。继续实施封滩育林、禁牧休牧、草原奖补、“三北”防护林工程，积极开展生态补偿实验区、荒漠化治理示范区和湿地补偿试点工作，完成人工造林12.7万亩，人工种草105万亩，义务植树358.8万株。深入实施“联村联户”和“1236”扶贫攻坚行动，落实扶贫资金2.97亿元，实施富民产业项目120个，输转移民乡村劳动力2.2万人，发展特色种植15万亩，调引基础母羊2.2万只，完成整村推进项目11个，改造移民住房2329户，移民乡村生产生活条件明显改善。

　　2、优化提升工业结构。规模以上工业完成增加值228亿元，增长17.8%。加快新能源首位产业发展，建成风电二期工程100万千瓦，65万千瓦大型风机示范项目并网发电，建成和在建风电装机1020万千瓦、光电装机3294兆瓦。积极创建国家新能源综合利用示范基地，京城控股发电机及电机制造、阳光电源逆变器、万晟光伏电池等产业链延伸项目建成投产，形成1.5兆瓦到5兆瓦风机整装年生产能力500万千瓦、成套光伏组件年生产能力300兆瓦，生产规模和市场占有率稳步扩大。调峰火电和电能外送通道建设取得重大突破，神华肃州电厂、瓜州常乐电厂等600万千瓦火电项目前期工作完成，累计建成火电装机88.3万千瓦、水电装机38.8万千瓦，发电量达174亿千瓦时，增长23.5%；敦煌至鱼卡750千伏第二通道电网工程建成投用，酒泉至株洲±800千伏直流输变电项目获批，8个330KV输电工程开工建设，风电企业平均上网率达到85%以上，实现全行业盈利。制定实施循环经济六个百亿元产业链建设方案，三新硅业扩建、恒亚水泥等一批循环经济项目快速推进，高载能产业集群不断壮大，电能消纳能力突破40亿度。制定特色工业园区建设方案和经济技术开发区发展指导意见，大力推进“一区多园”和“飞地建园”，酒泉工业园区升格为国家级经济技术开发区，酒泉现代资源综合利用产业园和敦煌工业园区创建为省级循环经济示范园区，玉门经济技术开发区确定为省级示范园区，全市各级园区入驻企业721户，吸纳就业6.2万人，实现工业增加值154亿元，占工业经济总量的85%。

　　3、加快发展第三产业。大力推进服务业综合改革试点和华夏文明传承创新区建设，完成第三产业增加值228亿元，增长12.5%。敦煌国际文化旅游名城建设确定为全省华夏文明传承创新区首位工程，敦煌国家级文化产业示范园区、酒泉生态文化旅游观光园、瓜州玄奘取经博物馆等138个文化旅游项目开工建设，3家企业进入全省民营文化企业30强；成功举办第六届丝绸之路国际大会暨第三届敦煌行·丝绸之路国际旅游节等活动，免费开放西汉酒泉胜迹景区，创建鸣沙山·月牙泉国家5A级景区通过初审，富康梦天堂、玉门赤金峡创建为国家4A级景区，全年接待游客1097万人次，实现旅游收入94.5亿元。酒泉精品商业街、汉唐美食街、敦煌文化商业一条街改造全面完成，高档宾馆饭店基本实现大众化消费转型。完成400个农家店信息化改造，全市基本实现商品配送中心、农贸市场、行政村三个全覆盖。加快区域物流中心建设，发展快递企业48家，巨龙现代农业物流港进入招商阶段，柳沟煤炭物流园转运吞吐能力达到1000万吨。建成省级以上工程技术研究中心和重点实验室12家、市级工程技术研究中心29个，生产性服务企业达到467户，吸纳从业人员1.1万人，实现营业收入70.4亿元。健全市域金融服务体系，浦发银行酒泉支行、甘肃银行酒泉分行挂牌成立，银行业金融机构达到18家；新增小额贷款公司10家、融资担保公司4家，分别达到49家和28家。组建工业、农业、文化、交通四大发展集团公司，举办第十届银政银企合作推介会，签约贷款金额434.2亿元，发放财政贴息贷款20.1亿元，增加小微企业贷款78.4亿元，直接融资达到25.8亿元。深入开展全民创业行动，新增私营企业、个体工商户、农民专业合作社等非公经济主体1.2万户，累计达到5.2万户，非公经济增加值达到310.5亿元，占GDP的48.5%，比上年提高2个百分点。

　　4、加速推进城镇建设。实施重点城镇建设工程275项，完成投资74.4亿元。坚持规划先行，注重用文化元素彰显城市特色，汉唐文化新区，北大河两岸土地整理、新城区二期、高铁片区等11个控制性详规批复实施，市区控规覆盖率达到95%。金塔、瓜州、肃北、阿克塞等县城总体规划修编完成，全市乡镇总体规划和村庄建设规划实现全覆盖，城镇化率达到54.5%，提高2.3个百分点。创新城市建设扁平化管理模式，风光大厦、酒文化博览园等既有建筑改造启动实施，完成新城区二期“一纵五横”道路管线及路基工程，河道景观及西一桥、人工湖、公园等附属工程加速推进，城市博物馆、飞天路景观建设完成年度任务。新建改造老城区文化路、敦煌路等11条道路和邮电街、西环北路等7条背街小巷，新增热电联供面积400万平方米、天然气用户2万户，鼓楼周边建筑全面美化，新增绿化面积117万平方米，市区人均公共绿地面积达到9.5平方米。各县市整体推进城镇基础设施和美化、亮化工程，城市形象和品位全面提升。加快市域交通体系建设，新建改造农村公路679公里，酒泉客运枢纽站和16个乡镇汽车站建成运营，连霍高速酒泉收费站扩建完工，肃阿二级公路全线通车，瓜敦快速通道完成部分路基工程，敦格铁路开工建设，兰新高铁酒泉段铺轨工程全部完成。大力推进新农村建设，新增农村安全用水居民5.2万人，硬化村组道路127条，改造农村危旧房1.3万户，建成村容村貌示范区31个，新建百户以上楼居式中心村26个，农村砖混砖木结构住房比重达到80%。酒泉第二污水处理厂项目启动，6个县市城区污水处理厂建成运行或开工建设，淘汰关停铁合金、小水泥等落后产能42.2万吨，单位工业增加值用水量下降7.5%，农村环境连片整治项目通过省级验收，节能降耗和污染减排完成省定目标。

　　5、着力保障改善民生。各级财政投入社会事业和民生保障资金57.4亿元，占财政总支出的51.4%。新增城镇就业3.4万人，落实就业补贴资金1.86亿元，新增创业贷款担保基金4208万元，发放创业及小额担保贷款3.25亿元，选拔扶持2136名高校毕业生到基层和企业就业。建立高龄老人生活补贴制度，城乡低保、企业退休人员养老金等社保待遇全面提标，平均增幅达15%以上，基本医疗保险、新农合和城乡居民社会养老保险参保率保持在98%以上。完善临时救助、慈善救助、灾害救助和大病医疗救助制度，落实低收入群体生活补贴与物价上涨联动机制，为城乡16.8万名困难群众发放生活补贴3992万元。新建城市社区日间照料中心12个、农村互助老人幸福院53个。新建保障性住房4253套，发放租赁补贴11460户，保障性住房建设任务全面完成，商品房价格涨幅控制在1.64%，低于省定目标。建立价格调控联动机制，落实肉类蔬菜价格一揽子调控措施，市场物价涨幅控制在3.5%。省级创新型试点城市启动建设，组织实施国家和省级科技项目78项，示范推广实用新技术180项，成立新能源研究院，国家种子加工装备工程技术研究中心落户我市。组建酒泉新能源学院，与兰州理工大学联合招收本科生123名，结束了酒泉没有全日制本科教育的历史；实施校舍安全工程、薄弱学校改造、农村幼儿园建设项目137个，新建校舍16万平方米，消除危旧校舍10万平方米，新建市级标准化学校65所，金塔、阿克塞列入全省率先实现义务教育均衡发展10县市之列。“营养改善计划”全面落实，惠及全市6.7万名农村中小学生。率先在全省实行县级医院基本药物、医用耗材和植入性材料“零差率”，农村急救中心、鼠疫应急物资储备库等卫生项目进展顺利，全科医生培训中心、精神病专科和儿童专科开工建设。认真落实计划生育优扶政策，扎实推进幸福家庭创建，孕前优生健康免费检查实现城乡全覆盖。5个县级“文化三馆”建成，锁阳城、悬泉置、玉门关河仓城申报世界文化遗产工作通过联合国专家组现场考察评估，广播电视“户户通”超额完成年度任务，成功举办“华夏文化艺术节”、第七届全市少数民族传统体育运动会、第十二届全运会沙滩排球资格赛、第九届哈萨克式摔跤亚洲锦标赛等文体活动。强化产品质量、食品药品和市场物价监管，确保了市场正常供应和群众饮食用药安全。深入开展“打非治违”和安全隐患排查专项行动，安全生产四项控制指标全面下降。完善突发公共事件应急预案，狠抓普法宣传、法律服务、人民调解、特殊人群管理和法律援助工作，积极开展“两访”问题“百日攻坚”、“初信初访提升年”和“信访积案化解年”活动，一批信访积案和群体性上访事件得到解决。深入推进“平安酒泉”建设，强化社会管理创新，肃州区、敦煌市市民服务中心建成运行。健全完善社会治安防控体系，新建视频监控点1540个，卡口30个，酒泉被命名为全国社会管理综合治理优秀市、全国平安铁路示范市和全国第十届见义勇为优秀城市。统计、人防、边防、供销、地震、气象、妇女、儿童、残疾人、台侨、民族、宗教、档案、史志、外事等工作取得新的成绩。

　　各位代表，一年来，我们认真落实中央“八项规定”、省委“双十条”和市委“十项规定”，健全为民、务实、清廉各项制度，各类庆典活动全部取消，楼堂馆所一律停建，办公用房清理全面完成，制定“三公”经费和一般性支出控制标准，“三公”经费下降15%以上。加强政府自身建设，完善决策议事规则，发展规划、重大项目、大额资金使用等重要事项一律提交政府常务会集体研究决定。自觉接受人大法律监督、工作监督和政协民主监督，邀请人大代表、政协委员列席市政府常务会议，办理人大代表意见建议和政协委员提案376件，办结率100%。推进法治政府建设，强化行政执法，健全规范性文件和重大决策合法性审查机制。深化审批制度改革，全面落实国务院、省政府取消下放的各类审批事项，取消合并下放市级行政审批项目144项。在全省率先建成行政审批电子监察系统和重大项目电子督察系统，推行限时办结和并联审批，办结时限压缩91.6%，行政服务中心成为全省首家通过标准化质量体系认证的公共管理服务单位。强化政府廉政建设，集中开展工程领域招投标、土地和矿产资源招拍挂、政府采购、财政资金使用管理、各项规定落实等领域专项检查，加强对预算执行、民生社保等专项资金和政府投资项目的审计监督。规范公共资源交易流程，保证了公共资源交易的公开公平公正。

　　各位代表，回顾一年来的工作，尽管经济形势复杂严峻，但我们自加压力，迎难而上，取得了转型发展的良好业绩，圆满完成了年初确定的预期目标。成绩的取得，是省委、省政府高度重视、亲切关怀的结果，是市委科学决策、正确领导的结果，是市人大、市政协和社会各界大力支持、有效监督的结果，是全市人民凝心聚力、砥砺奋进的结果。在此，我代表市政府，向各位人大代表、政协委员和广大干部群众，向各民主党派、工商联、人民团体、离退休老同志，向驻酒解放军指战员、武警官兵和公安干警，向中央、省属驻酒单位以及所有关心、支持酒泉发展的各界人士，致以崇高的敬意和衷心的感谢！

　　在总结成绩的同时，我们也清醒地看到，经济社会发展还面临一些困难和问题，突出表现在：一是受央企业绩考核方式改变和电力输出不畅、融资困难等因素制约，部分新能源项目没有实现进度目标。二是项目审批环节多、落地难的问题还没有得到根本改善。三是招商引资针对性不强，引进项目中资源开发和初加工项目居多，精深加工项目较少。四是现代服务业发展缓慢，社会消费对经济增长的拉动力不强。五是支柱产业和主体税源发展不匹配，税收增长不足，财政保障压力较大。六是扶贫攻坚任务艰巨，移民乡村及沿山片区发展落后，改善基础设施条件、大幅增加收入的难度较大。七是政府工作效率与人民群众的愿望和要求还有一定差距，机关效能建设还需要进一步加强。对此，我们将在今后的工作中认真研究解决。

　　2014年工作总体部署

　　2014年是全面落实党的十八大和十八届三中全会精神的重要一年，也是实现“十二五”规划的关键一年，加快经济社会发展既有许多有利因素，也面临着多方面的挑战和压力。中央全面深化改革的部署，突出市场对资源配置的决定性作用，宏观政策对“三农”、社会事业、保障性住房、中西部地区交通基础设施建设给予倾斜支持，尤其是启动“丝绸之路经济带”战略，实施华夏文明传承创新区和国家生态安全屏障实验区建设，为我们扩大投资、加快转型提供了难得的机遇。同时，区域发展竞争日趋激烈，经济下行压力依然存在，发展中的不确定因素较多。我们必须以对党和人民高度负责的态度，增强责任感、使命感和紧迫感，以奋发有为、锐意进取的精神，加快推进酒泉经济社会全面协调可持续发展。

　　2014年政府工作的总体要求是：以党的十八大和十八届三中全会精神为指导，深入贯彻省第十二次党代会和市委三届四次、五次全委会议精神，稳中求进、改革创新，继续按照“五做五加强”总体工作思路，加快实施“3341”项目工程，着力打造发展战略平台，着力营造干部清正、政府清廉、政治清明的良好发展环境，全力推动经济社会转型跨越发展。

　　全市经济社会发展的预期目标是：地区生产总值增长12%以上，固定资产投资增长25%以上，社会消费品零售总额增长15%以上，公共财政预算收入增长15%以上，城镇居民人均可支配收入增长13%以上，农民人均纯收入增长14%以上，万元生产总值能耗降低3%，主要污染物排放完成约束性指标。重点抓好七个方面的工作：

　　1、聚焦薄弱环节，深化重点领域改革。进一步深化市情认识，重新审视酒泉的发展定位，认真学习研判国家宏观政策，积极争取优惠政策，探索推动转型跨越的体制机制，全面落实各项改革举措，着力破解发展难题。制定更加优惠的政策措施，全力支持非公经济参与基础设施、产业发展和社会事业，做强做大产业规模，力争非公经济占GDP比重提高2个百分点以上。完善金融激励考评机制，健全金融服务体系，促进融资发展集团规范运行，着力放大融资规模，培育和支持本地企业多渠道融资上市，直接融资增长25%以上。完善酒泉经济技术开发区“一区七园”管理体制，理顺财税管理和利益分成办法，落实“飞地建园”和跨县区招商项目分享激励政策。合理确定市区事权和财权，激发各级发展活力。完善部门预算公开、专项资金使用和财政转移支付监督机制，保证资金安全、高效使用。

　　2、狠抓项目建设，夯实转型跨越基础。以“3341”项目工程为引领，紧盯国家投资导向和东中部产业转移，找准政策与产业的结合点，抓好项目谋划和论证，合理布局产业发展、基础设施、生态环境和社会民生项目，加强政府性投资项目预算管理、资金投放和组织实施。加快瓜敦快速通道和敦格铁路建设，争取敦当高速、白明高速、酒航高等级公路、公铁联运站开工，做好酒额铁路前期工作，按期完成高铁酒泉南站建设，确保兰新高铁顺利通车。积极争取生态安全屏障综合试验区项目，启动实施河西走廊高效节水和以色列政府贷款项目，继续抓好灌区续建配套、病险水闸加固、河道治理工程，加快敦煌水资源利用与生态保护规划项目特别是引哈济党工程建设进度。做好生态文明建设示范区、防沙治沙示范区和沙化土地封禁保护试点工作，继续实施封滩育林、生态公益林、禁牧休牧、退牧还草、“三北”五期防护林和湿地保护等生态修复工程。完善领导干部和部门招商责任，研究分析三大产业各自优势产业链条的产业构成，瞄准世界500强、国内500强和民营500强企业，着力引进产业配套项目、资源综合利用项目和高附加值项目，提高引资项目的针对性和目的性。

　　3、坚持做精一产，力促农民稳步增收。加快构建新型农业经营体系，扩大土地确权登记颁证试点，赋予农民更多的财产权利，支持专业大户、家庭农场、农民合作社、农业企业等经营主体发展现代高效节水农业，探索建立土地流转交易市场，土地流转率提高4个百分点。深化水权水价改革，落实差别水价政策，探索开展水权交易试点，全市用水总量控制在30亿立方米以内，用水效率和水功能区限制纳污控制在省上下达的指标以内。认真落实强农惠农政策，着力提升肃州国家级和敦煌省级现代农业示范园区建设水平，力争农业增加值增长6%以上。加强农业服务体系建设，积极推进农业标准化生产和规模化发展，围绕高效蔬菜、现代制种、优质林果、特色中药材和草食畜牧业五大优势产业，加快发展精深加工，农产品加工增值率达到60%以上。突出县域特色，建设标准化、规模化生产基地，特色种植面积增长7%以上，牛羊规模饲养量增长8%以上。坚定不移地发展设施农业，强化专业化分工，提高各生产环节对农民增收的贡献率。健全完善农产品质量监测体系和动物疫情防控体系，确保农产品合格率达到99%以上。坚持打好移民扶贫攻坚战，积极争取扶贫政策和扶持资金，整片整村推进移民乡村基础设施建设和增收产业培育，力争移民人均纯收入增长25%以上，贫困人口减少30%以上。

　　4、着力做强二产，推动工业转型升级。突出市场在资源配置中的决定性作用，编制实施工业发展规划，制定培育壮大首位产业、六个百亿元产业链和改造提升传统产业实施方案，合理布局产业项目，落实扶持政策，支持地方企业进入股权市场和生产资料及工业品交易市场，做粗做长产业链条，力争工业增加值增长14.5%以上。加快新能源首位产业发展，建成风电二期首批300万千瓦项目，争取后续500万千瓦项目开工建设；新增光电装机100万千瓦，做好光电二期300万千瓦发展规划，风、光发电量增长17.7%以上，力争进入全国新能源示范城市；重点引进短板补链项目和高端制造项目，推进新能源技术研发与装备制造、风光电场建设融合发展，争取国家风电研究检测中心测试基地、太阳能光伏发电系统质量检测中心实验室落户酒泉，首位产业占工业增加值比重达到35%以上。做好酒泉热电厂二期、昌马120万千瓦抽水蓄能电站等项目前期工作，争取开工建设瓜州常乐电厂和肃州电厂，提高电源平衡能力。开工建设酒泉至株洲±800千伏直流输变电工程，争取酒泉至川渝地区特高压直流外送工程列入国家规划。加大循环经济产业建设力度，扶持三新硅业、海源矿业等优势企业扩张规模，全面建成永鑫科技、万晟光电等硅材料加工项目，支持玉门油田争取炼化扩容和石油储备库建设，力促广汇千万吨级煤炭分质利用项目开工建设，力争循环经济产业占工业增加值的比重达到45%以上。积极推进传统产业集群发展，引导龙头企业组建产业发展联盟，扶持小微企业做大做强，力争规上企业增加15%以上，小微企业增加20%以上。落实国家级经济技术开发区建设发展方案，加强园区基础设施建设，着力增强园区聚集承载能力。

　　5、加快做大三产，提升服务业发展水平。深入推进服务业综合改革试点，突出文化旅游业的龙头作用，力争第三产业增加值增长10%以上。引进做强广汇、金轮、凯泰置业等大型现代物流企业，扶持壮大瓜州柳沟、玉疆捷达等专业物流园区，整合规范现有生产资料、建筑建材等销售市场，新建一批专业批发市场和综合性商业区、特色商业街，活跃城乡消费市场。大力发展文化创意等新型服务业态，提升金融消费、网络购物、社区托幼、家政保健等便民服务业层次。规范中介机构从业行为，切实提高行业整体素质。大力推进酒泉汉唐文化新区、敦煌国际文化旅游名城建设，抓好敦煌国家级文化产业示范园区、旅游综合改革试验区、文化生态保护区、生态文明建设示范区申报工作，加快夜光杯博览园、丝绸之路主题文化园等百项千亿华夏文明保护传承创新区建设项目，力争文化产业增加值增长45%以上，占GDP比重达到2.2%以上。整合文化旅游资源，创新运营管理机制，提升景区服务设施配套功能，加快旅游产品提档升级，加强冬春旅游市场开发，力争游客接待人数和旅游收入双双增长20%以上。加强与“丝绸之路经济带”沿线国家和省区市的交流合作，规划建设丝绸之路国际商贸城，办好国家新能源产业博览会、第四届敦煌行·丝绸之路国际旅游节等节会，争取承办丝绸之路国际商品博览会、丝绸之路·敦煌国际文化博览会，努力将酒泉打造成为“丝绸之路经济带”上的重要战略通道和区域中心城市。加强海关、商检和口岸建设，做好敦煌航空口岸开放和马鬃山口岸复关工作，进一步提高经济发展的外向度。

　　6、立足做美城市，提高综合承载能力。加快推进以人为核心的城镇化建设，完善城市功能，提升城市品位，彰显城市特色。坚持科学规划、突出特色，做好酒嘉新区等6个片区控制性详规编制，开展新型城镇化发展规划编制工作，以城乡一体化为目标，完善城市综合交通、供排水、公共服务设施、土地利用等专项规划，实现城乡规划全覆盖。加快实施汉唐文化新区、北大河生态环境综合治理、新城区二期开发和高铁片区建设，配套完善水电气热、垃圾污水处理等基础设施，初步形成新城区二期路网框架；加快老城区市政设施改造和维护力度，新建改造一批健身休闲娱乐场所，进一步增强城市服务功能。加强生态园林建设，启动全国园林城市、国家卫生城市、国家环保模范城市和全国文明城市创建工作，改善城市生态环境。加快智慧城市建设，构建“数字酒泉”地理空间框架，提高数字化城市管理运行水平。强化城市综合执法，提升市民素质，深入开展市容市貌、交通秩序、市场秩序整治，不断提升城市形象。加快城镇市政设施建设，完善教育、医疗、文化、商贸等公共服务设施，推进城乡要素平等交换和公共资源均衡配置。全面放开建制镇和小城市落户限制，有序推动农村转移人口市民化，力争城镇化率提高1.5个百分点。

　　7、全力做实民生，增进人民群众福祉。从群众最关心最直接最现实的利益出发，努力为社会提供多样化服务，让发展成果更多更公平惠及全体人民。健全促进就业创业体制机制，落实技能培训、小额贷款、财政补贴等就业创业服务措施，实施千名高校毕业生企业就业计划，城镇登记失业率控制在4%以内。建立健全社会救助制度，完善社会保障预算制度和社会养老服务体系，城乡居民养老保险和医疗保险覆盖面巩固在98%以上。积极推进科技创新体系建设，抓好省级创新型城市试点工作，力争科技进步对全市经济社会发展的贡献率达到54%以上，把酒泉创建为国家知识产权试点城市。深化教育综合改革，健全城乡教育一体化发展管理体系和资源配置机制，加快推进薄弱学校改造、公办学校标准化建设和校长教师交流轮岗，探索建立职业教育与园区企业的有效对接模式。深化文化体制改革，鼓励社会资本参与公共文化服务体系建设，深入推进公益性文化事业单位内部人事和收入分配制度改革，推动公共文化服务设施免费开放。大力实施体育惠民工程，办好群众体育品牌赛事，积极组织参加全省、全国性体育赛事活动。深化医药卫生体制改革，推进政事分开、管办分开、医药分开、营利性与非营利性分开，完善公立医院合理补偿机制，建立新农合医疗风险金市级统一管理制度。启动实施单独两孩生育政策，人口自然增长率控制在5.5‰以内。强化价格监管，确保生活必需品供求基本平衡，居民消费价格涨幅控制在4%以内。广泛开展法制宣传教育，积极实施法律援助。深入开展双拥共建活动，扎实做好优抚安置工作，力争新一轮省级双拥模范城创建通过验收。继续做好妇女儿童、老龄、残疾人、外事、侨务、民族、宗教、统计、供销、地震、史志、人防、档案、慈善、红十字会等工作。

　　切实解决民生问题，继续推进热电联供和天然气入户工程，办好十件实事：一是继续实施校舍安全工程，建设农村寄宿制学校标准化食堂20个；二是新建改建农村公路600公里，实施移民贫困村整村推进项目10个；三是实施市医院精神病区、儿科病区、全科医生临床培养基地和三级医院综合服务能力建设工程，完成乡镇卫生院及村卫生室改扩建年度任务；四是开展“关爱万名贫困母亲”行动，救助贫困母亲1万名；五是解决5万名农村居民饮水安全问题；六是实施城区自备水源井关停并网工程，提高1.3万名酒泉城区居民饮水质量；七是组织开展“图书漂流”服务活动，在景区、社区、宾馆、学校设立服务点25个；八是新增各类保障性住房4600套，发放租赁补贴7900户；九是继续组织实施“天眼百卡”工程，新建高清视频监控点1000个，智能卡口20个；十是新建惠民肉菜直销店30个。

　　努力建设人民满意政府

　　全面深化改革对政府工作提出了更高的要求。面对新的形势，担当挑大梁、做标杆、走前列的重任，必须弘扬敢为、能为、有为的精神，努力建设服务型、效能型和廉洁型政府。

　　一是转变政府职能，建设服务型政府。强化公共服务、市场监管、社会管理、环境保护等职责，深化行政审批制度改革，让市场发挥配置资源的决定性作用，最大限度地取消和下放市级行政审批事项，规范办事程序，更加重视在制度机制上规范市场秩序，严格落实安全生产、环境保护、食品药品监管责任，确保人民群众生命财产安全。进一步改进社会治理方式，注重网络舆情的研判、引导和处置，畅通群众诉求渠道，构建社会治安立体防控体系，努力实现公共服务均等化和社会管理的科学化、制度化、规范化，真正把该管的管起来，该放的放到位。

　　二是改进机关作风，建设效能型政府。严格落实中央“八项规定”、省委“双十条”和市委“十项规定”，深入开展党的群众路线教育实践活动，倾听群众呼声，关心群众疾苦，多做解民忧、惠民生的事，多做打基础、利长远的事。强化限时办结和并联审批制度，优化工作流程。加快推进企业工商注册制度便利化，建立政务管理信息资源共享平台和电子政务统一网络，不断提高政府效能。

　　三是狠抓反腐倡廉，建设廉洁型政府。认真执行党风廉政建设责任制，严格落实“一岗双责”，创新廉政风险防控机制，加快形成不敢腐的惩戒机制、不能腐的防范机制、不易腐的保障机制。全面贯彻《党政机关厉行节约反对浪费条例》，严格落实政府性楼堂馆所不得新建、财政供养人员只减不增、三公经费支出压减的“约法三章”规定，把有限的资金更多地用在改善民生上。严格依法行政，始终按照法定程序行使权力，自觉接受人大法律监督、工作监督和政协民主监督，高效办理人大代表意见建议和政协委员提案。加强行政监察和审计监督，启动建设住房公积金、社保资金电子监察系统，加大工程建设、土地出让、政府采购、征地拆迁等重点领域监管力度，用制度管权、靠制度管人、按制度办事。

　　各位代表，新的一年，新的征程。让我们在市委的坚强领导下，勇于担当，锐意进取，埋头苦干，奋力谱写建设富裕文明和谐幸福酒泉的新篇章。
